# Psychological Impact on the Orthodontic Postgraduate Residents and Their Anxiety Level during the COVID-19 Pandemic

**DOI:** 10.1155/2022/3143475

**Published:** 2022-12-31

**Authors:** Hareem Sultan, Sameeruddin Shaikh, Sadaf Shaheen, Hana Pervez, Syed Adnan Ali, Saman Baseer

**Affiliations:** ^1^Dow International Dental College, Dow University of Health Sciences, Mehmoodabad Road, Karachi, Pakistan; ^2^Rashid Latif Medical and Dental College, Lahore, Pakistan; ^3^Jinnah Medical and Dental College, Karachi, Pakistan; ^4^Government Degree Science and Commerce College, Karachi, Pakistan; ^5^Sardar Begum Dental College, Gandhara University, Peshawar, Pakistan

## Abstract

**Background:**

COVID-19, a viral infection, has negatively impacted the physical and psychological health of the people worldwide. This was a descriptive, cross-sectional study, which aimed to investigate orthodontic postgraduate residents' knowledge regarding COVID-19, its association with anxiety around quality of their training, and performance of routine orthodontic procedures during the pandemic. A questionnaire was circulated online through WhatsApp to the orthodontic residents enrolled in the four-year postgraduate training program across the country which comprised the following sections: demographic data, questions to test knowledge of COVID-19, self-grading of anxiety around performing routine orthodontic procedures, academic aspects of the training program, and residents' thoughts on the current status of their training program in general during the pandemic. The residents were grouped based on their awareness on COVID-19, with >70% score taken as having adequate knowledge and ≤70% as inadequately updated. The association between COVID-19-related knowledge and the anxiety level of the residents experienced during training was assessed. Results were analyzed using SPSS (Statistical Package for Social Sciences) version 23.0. Counts and percentages were reported on baseline characteristics of studied samples. Descriptive methods were used to report the outcomes of this survey on knowledge of COVID-19, anxiety around performing orthodontic procedures, and different academic aspects of training. Statistical significance was set at *p* < 0.05 using the Pearson chi square test.

**Results:**

Most of the residents (51.5%) were adequately updated about the COVID-19 virus. Increased awareness was associated with more anxiety about the following: contracting the virus (*p* < 0.01), performing different orthodontic procedures (*p* < 0.05), disturbed patients' appointments (*p* < 0.01), timely completion of their cases (*p* < 0.01), and exam's preparation (*p*=0.04). The group with <70% COVID-19 related knowledge opted for extension of their training period (51.8%).

**Conclusion:**

COVID-19 related knowledge was clearly associated with anxiety experienced by the orthodontic residents during training in the pandemic. Awareness regarding the infection led to more anxiety around working during the pandemic, preparing for postgraduate exams, and concerns about its negative influence on the overall quality of the training program.

## 1. Introduction

COVID-19 was recognized in December, 2019, and has since been the latest threat to global health, causing coronavirus disease worldwide [1]. It manifests as fever, cough, shortness of breath, pneumonia, gastrointestinal symptoms, and acute respiratory distress syndrome leading to multiple organ failure [2].

COVID-19 spreads via inhalation of droplets from an infected person in close contact (<6 feet). This makes the dental community most prone to contract the disease, as they work in close proximity to their patients. This led to a widespread use of N95/FFP2 respirators that had their own side-effects [3]. Its incubation period is five days with 97.5% of infected subjects developing symptoms within 11 days, which means dentists could be treating asymptomatic COVID-19 patients at the cost of risking exposure to the disease and spreading it exponentially amongst the people in close contact [4].

Besides affecting physical health, COVID-19 has been the leading cause of widespread anxiety and psychological illness as well, since January, 2020 [5]. According to Encyclopedia of Psychology, anxiety is an emotion characterized by feelings of tension, worried thoughts, and physical changes like increased blood pressure. Furthermore, health anxiety is characterized by: distressing emotions, bodily sensations, thoughts and images of danger, avoidance, and other defensive behaviors [6]. The constant flow of information regarding the pandemic, reminders regarding death and unpredictability of health status, imposition of lockdowns keeping people homebound, and uncertainty in income stability has affected populations' mental health worldwide [7]. The first study on the mental wellbeing of the residents was done by Alhaj et al. on neurosurgery residents and 90% of the residents were found to be affected due to the pandemic [8]. Shortage of personal protective equipment and prolonged working hours made it all the more taxing on mental wellbeing and physical health of the healthcare workers [9]. Knowledge and education regarding infectious diseases could positively influence the outlook and attitude of dentists when treating patients with infectious diseases; whereas, contradictory evidence had also been reported [10]. Postgraduate residents were anxious regarding the limited exposure to operative procedures, maintaining clinical competence, and risking their families' health, which was even more worrisome and eventually led to burnout [11]. On the contrary, 66.34% of the respondents reported improved social life due to more free time, thanks to the lockdown! [12].

The literature lacks evidence on the consequences of COVID-19 pandemic's restrictions on the postgraduate residents belonging to the discipline of orthodontics. Success in the orthodontic postgraduate exam requires thorough knowledge of the subject along with completion and presentation of five orthodontic cases finished to the highest degree of precision and accuracy. Therefore, the orthodontic trainees are required to complete the cases in time in order to sit the exams. It becomes overbearing as their own vigilance in addition to their patients' compliance influences the ultimate success of the finished cases. Our study aims to assess the orthodontic residents' knowledge and awareness related to the COVID-19 virus, its association with the anxiety around performing routine orthodontic procedures, and impact of the pandemic on the overall quality of the training program.

## 2. Materials and Methods

This research was a questionnaire-based descriptive cross-sectional study carried out on orthodontic residents/trainees enrolled in the four-year postgraduate training program.

### 2.1. Ethical Approval

The research proposal was approved by the ethical review committee of de ‘Montmorency College of Dentistry, Lahore, Pakistan (Number 306, Dated 06-02-2021).

### 2.2. Inclusion Criteria

The inclusion criteria were as follows: postgraduate residents who enrolled in the four-year training program across the country, those who were accessible through WhatsApp, consented to participate in the study, were able to read and comprehend English language, had access to Google Forms, and those who could fill and submit their responses online, successfully.

### 2.3. Data Collection

The number of orthodontic residents across the country enrolled in the training program was about 330 at that time. The study's questionnaire was first circulated among 20 residents for a pilot survey to ensure clarity and interpretation of the questions and determine the time required for its completion. The actual survey started after getting the responses from the pilot study. Data were collected from 15^th^ March, 2021, to 15^th^ May, 2021. Three reminders were given to the residents after every two weeks. Completion and submission of the questionnaires by the participants was taken as the implied consent. A total of 171 (52%) responses were received. Sample size was not calculated as this study aimed to reach maximum number of postgraduate residents being trained during the specified time period.

### 2.4. Questionnaire

The questionnaire was made on Google Forms and circulated online through WhatsApp groups (of residents) and sent privately too. The study's questionnaire comprised questions related to demographics (without mentioning the participants' names and keeping their data anonymized); knowledge about COVID-19 virus/infection, which was followed by a section on self-grading of anxiety experienced while performing different orthodontic procedures and the residents' thoughts around other academic aspects of training. The section on COVID-19-related knowledge comprised basic questions on etiology, incubation period, isolation period, social distancing required, route of transmission, symptoms, diagnostic tests available, and currently available treatment options for the COVID-19 infection and PPE (personal protective equipment) recommendation for healthcare workers.

Participants' knowledge was assessed and marked by matching their answers with those of CDC (Centers for Disease Control and prevention) guidelines at that time. Cutoff was taken to be 70% with those scoring more than 70% in this section being considered as adequately updated on COVID-19-related information. The section assessing the residents' anxiety consisted of fifteen questions, which required them to self-grade their anxiety on a scale of 1 to 5; 1-not anxious (NA), 2-slightly anxious (SA), 3-fairly anxious (FA), 4-very anxious (VA), and 5-extremely anxious (EA). The procedures included were as follows: changing of ligatures, insertion of arch wires, etching with washing and drying using triple syringe and suction, cementation of orthodontic bands, bonding of orthodontic brackets, recording of impressions, taking photographic records, removal of orthodontic bands, debonding of brackets using hand instruments or high speed hand pieces, insertion of separators, removal and insertion of removable appliances, consultation of the new patients visiting the OPD, anxiety experienced during observing the procedures, and anxiety experienced during performing the procedures in general. Moving forward, the next section comprised questions related to the anxiety experienced by the residents around other aspects of training, such as risking their own and their families' health by working during the pandemic, disturbed patients' flow and lockdown restrictions impacting the duration and quality of orthodontic treatment of the cases, fear of patients quitting their treatment in between, and their preparation for the final postgraduate (fellowship) exam. The last section of the survey aimed to assess the residents' thoughts regarding the status of their training and how the pandemic had affected the educational activities and clinical competence that form an essential part of any postgraduate training program. The questionnaire could only be submitted once all the questions had been answered and marked. Once submitted, the responses could not be changed or omitted. The submitted responses were downloaded and exported through Microsoft Excel, 2013.

### 2.5. Statistical Analysis

This cross-sectional study was analyzed using SPSS (Statistical Package for Social Sciences) version 23.0, after inserting the responses obtained from the online survey. Counts and percentages were reported on baseline characteristics of studied samples, which included information on age group, gender, province, training institution, year of residency, and confidence of having knowledge on COVID-19. Descriptive methods were used to report the outcomes of this survey on knowledge of COVID-19, diagnostic tests, treatments, PPE, and anxiety related to performing routine orthodontic procedures during the COVID-19 pandemic.

## 3. Results

Out of nearly a total of 330 residents, 171 submitted their responses through the online forms.

Table 1 reports the baseline characteristics of studied samples with respect to COVID-19 knowledge scores. Results showed that majority of the (51.5%) residents scored >70% in the section related to knowledge about COVID-19. In samples found with ≤70% knowledge of COVID-19, 50.6% considered themselves to be updated about COVID-19. In samples with >70% COVID-19 knowledge (52.3%), 67% considered themselves to be updated about COVID-19. However, the Pearson chi square test did not give any significant association of COVID-19 knowledge with the baseline characteristics (*p* > 0.05).

Table 2 shows the responses of the participants to questions on knowledge regarding COVID-19, which allowed marking of a single option only, where most of the participants responded correctly.

**Figure 1:**
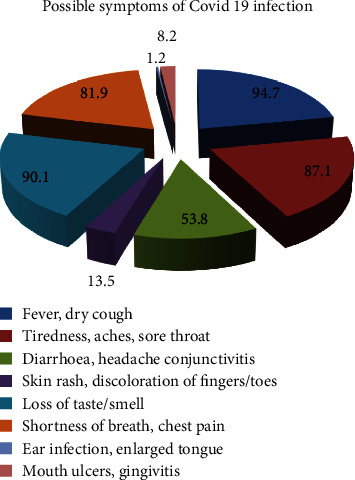
Percentage of each response as marked by the residents when asked about the possible symptoms of COVID-19 infection.

Figure 1 shows the responses of the participants to the question regarding possible symptoms of the COVID-19 infection. The participants could mark multiple options in this question. Significant number of responses (94.7%) marked fever and dry cough. It was evident that in all the options given, most of the correct responses were from the group with >70% of COVID-19 related knowledge.

**Figure 2:**
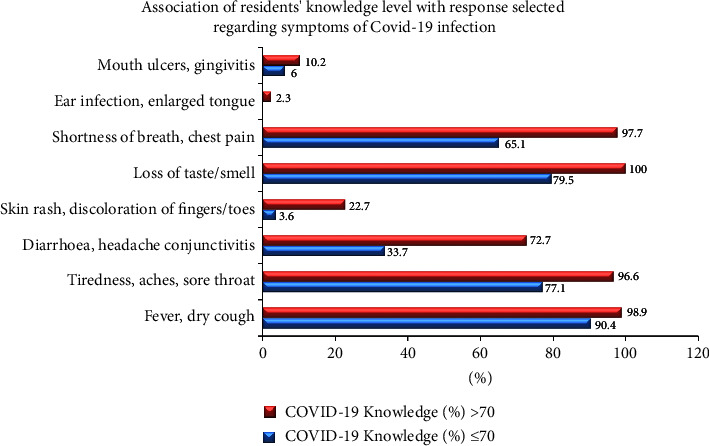
Association of level of COVID-19-related knowledge of the residents with their response selected regarding the symptoms of COVID-19 infection.

Figure 2 shows that among samples with higher COVID-19 knowledge (98.9%) marked fever, dry cough as possible symptoms of COVID-19.

Table 3 shows the outcomes on the questions related to the tests available for diagnosis of COVID-19 infection, its treatment options present till date, and PPE recommendation by WHO (World Health Organization) for the healthcare frontline workers. Multiple options could be selected in this part of the questionnaire too. The most popular diagnostic test amongst the participants was RT-PCR (real-time polymerase chain reaction), 90.6% responses. In PPE recommendation by WHO, face shields received the highest number of responses (94.2%) followed closely by gloves (93%). The results suggested adequate knowledge of residents.

**Figure 3:**
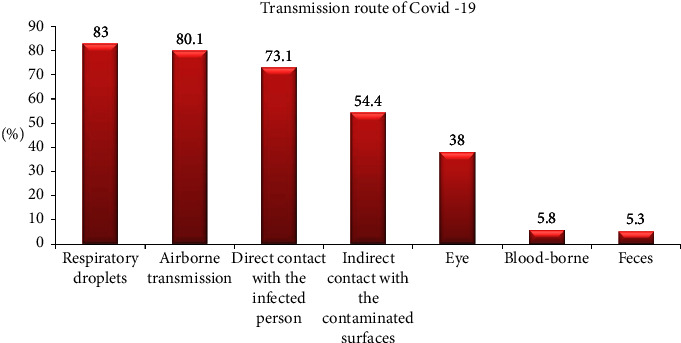
Percentage of the responses marked by the residents regarding the transmission route of COVID-19 infection.

**Figure 4:**
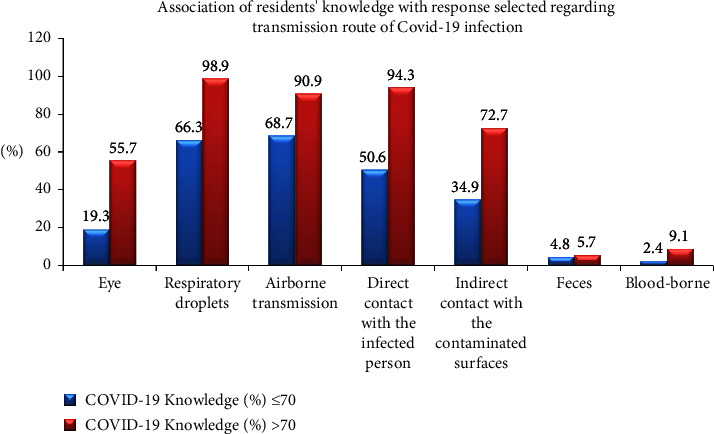
Association of COVID-19-related knowledge of the residents with their responses on the transmission route of COVID-19 infection.

Figure 3 reports the responses on transmission route of COVID-19, where 83% of the participants responded by selecting respiratory droplets, followed by airborne transmission (80.1%). This question allowed marking of multiple options. An association was found between the responses selected by the more updated group when compared to their counterparts, as shown in Figure 4, among samples with higher COVID-19 knowledge, 98.9% marked respiratory droplets as the transmission route of COVID-19 as compared to 66.3% in the group with <70% knowledge on COVID-19.

Table 4 reports the association between anxiety experienced by the orthodontic residents while performing routine orthodontic procedures and their knowledge regarding COVID-19. A significant association was observed while performing these procedures: (1) etching, washing, and drying with triple syringe and suction, (2) bonding of orthodontic brackets, (3) debonding using hand piece to remove the adhesive, and (4) consultation (taking history and doing clinical examination) of new patients visiting the orthodontic OPD (out patient department), (*p* < 0.05).

Table 5 reports the association between COVID-19-related knowledge and the anxiety experienced by the orthodontic residents when asked about observing or performing orthodontic procedures, risk of contracting COVID-19 infection from the OPDs, disturbance in patients' appointments leading to prolonged treatment, fear of patients' quitting their treatment prematurely, and pressure of timely completion of cases in order to sit the postgraduate exam. A significant association of anxiety was found around: (1) performing the dental procedures themselves, (2) possibility of contracting the COVID-19 infection from their institutes' OPD and risking their own and their family's health, (3) disturbed patients' appointments due to lockdown, and (4) pressure of completing their orthodontic cases in time in order to appear for the final postgraduation (*p* < 0.05).

Table 6 shows the relation between COVID-19-related knowledge and the anxiety around different academic and curricular activities essential for the residents to prepare them for the postgraduate exam. It reports, among samples with ≤70% COVID-19 knowledge, mostly, 61.4% reported anxiety causing disturbance in study schedule; less than half of the residents 38.6% thought social distancing jeopardized group study activity with other residents. Most of the residents agreed that conducting routine journal clubs, case presentations, and lectures became difficult and worrisome. Less than a half thought restrictions on national and international workshops/seminars led to limited learning experience and exposure, affecting their preparation for the final postgraduate exam and a good number was shown to be inclined towards having online academic activities during this pandemic to minimize close human contacts. A significant association was observed between residents' preparation for the final postgraduate exam with level of COVID-19 knowledge, (*p* < 0.05).

**Figure 5:**
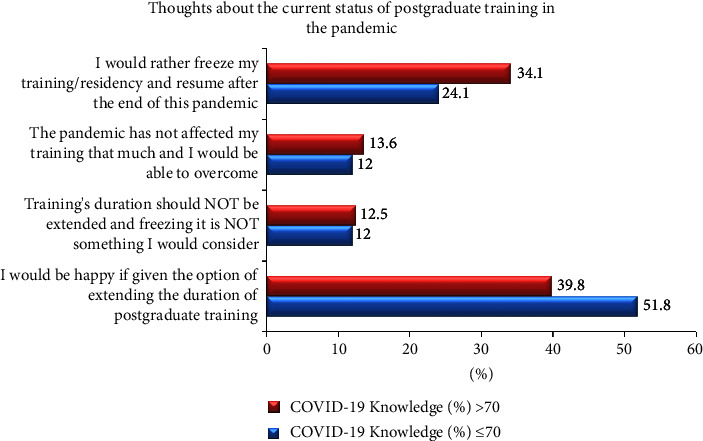
Association of the level of COVID-19-related knowledge with the thoughts of the residents on status of their postgraduate training during the pandemic.

Figure 5 shows the orthodontic residents' thoughts on the current status of their training in the pandemic. This question allowed a single response only. Results clearly showed that majority of the residents would opt for extending the duration of their training, if given the option, most of them were in the group with ≤70% knowledge. This was followed closely by 34.1%, (>70% COVID-19-related knowledge group) who were inclined toward freezing their training and resuming postpandemic versus 24.1% in the other group who also opted for the latter.

**Figure 6:**
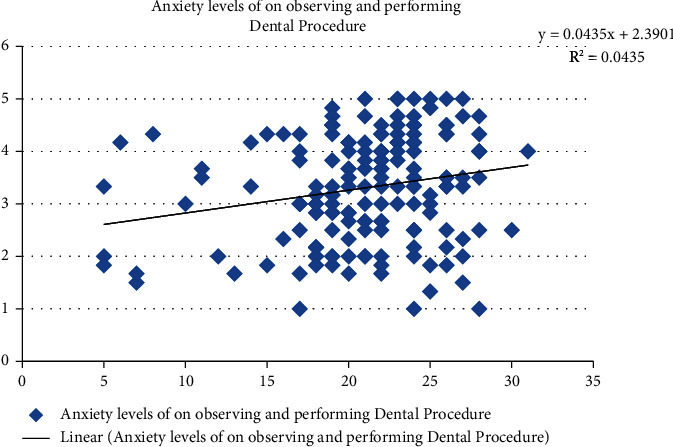
Association of COVID-19-related knowledge scores and anxiety level of the residents while observing and performing routine orthodontic procedures.

Figure 6 shows a positive association in COVID-19-related knowledge scores and anxiety level associated with observing and performing routine orthodontic procedures. R-square showed a 4.4% variation in anxiety levels which was explained by the COVID-19 knowledge scores.

**Figure 7:**
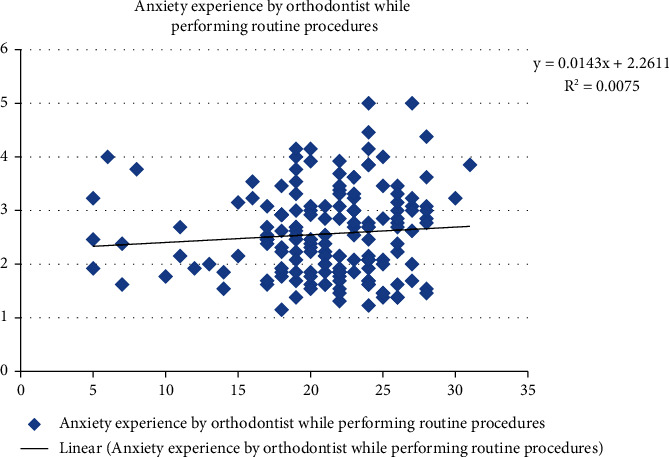
Association of COVID-19 related knowledge scores and anxiety experienced by orthodontic residents while performing routine orthodontic procedures.

Figure 7 shows a positive association in COVID-19-related knowledge scores and anxiety experienced by orthodontic residents while performing routine orthodontic procedures. R-square showed a 0.7% variation in anxiety experienced by orthodontic residents which was explained by the COVID-19 knowledge scores.

Figure 5 shows that among samples with higher COVID-19 knowledge, 39.8% thought they would be happy if given the option of extending the duration of their postgraduate training.

## 4. Discussion

This survey was conducted in an underdeveloped country, where the first case of COVID-19 was reported in February, 2020, [13] and since then a continuous spread had been reported across the country. In March, 2021 (when this study's questionnaire was circulated amongst the residents across the country), the latter was facing the third wave of COVID-19, which was specifically marked by increased infectivity rates as compared to the previous two waves of the infection. It peaked in late April, 2021. Nationwide lockdown was imposed in the first week of April, “2021, which extended till 9th May,” 2021, after which it was eased in phases and “smart lockdowns” were imposed [14]. Till date, 45% of the country's population has been fully vaccinated and 12% partly vaccinated against COVID-19 [15].

To assess the association between residents' COVID-19-related knowledge with the anxiety experienced during training, the respondents were grouped into two—residents with ≤70% COVID-19-related knowledge and those with >70% COVID-19-related knowledge. In both the groups, the residents were mostly females, within the age range, 20–29 years, in the fourth (final) year of their residency, being trained at public training institutes in the province of Punjab and most of them being confident about being updated on COVID-19. This pattern could be explained by Punjab being the most densely populated province of the country [16]. It has greater number of training institutes offering postgraduate training in the discipline of orthodontics [17].

This study showed that most of the residents were well aware of the COVID-19 infection and its characteristics. When asked about the possible symptoms of COVID-19 infection, most of the residents responded correctly [18]. All the residents with >70% COVID-19-related knowledge selected, loss of taste or smell as possible symptoms of the COVID-19 infection. Interestingly, some respondents in the latter group also marked the incorrect options-ear infections/enlarged tongue, 2.3%, whereas none of the respondents in the group with <70% knowledge about COVID-19 marked the latter.

Residents showed awareness regarding diagnostic tests for COVID-19, its available treatment options, and the PPE recommendation by WHO for the healthcare workers. This was a good sign that the orthodontic residents were well aware of the cross infection control measures to contain the spread of the contagious virus, as evident in one of the researches where people updated about the infection control measures showed better compliance with the guidelines [19]. The question about the route of transmission of the COVID-19 was also answered satisfactorily by most of the residents. On the contrary, 80.2% of the respondents in China (when enquired about COVID-19-related information) were confident about themselves being updated; however, majority of them were only able to answer less than half of the questions correctly [20].

This study also assessed the residents' anxiety while performing routine orthodontic procedures in the pandemic where the imminent danger of contracting the virus was very high. Four out of thirteen orthodontic procedures showed significant results, which were etching, drying with triple syringe, and suction (*p* < 0.01), bonding of orthodontic brackets (*p* = 0.03), debonding of orthodontic brackets using hand piece for removal of the adhesive (*p* < 0.01), and consultation of new patients visiting the orthodontic OPD (*p* = 0.02). The group with >70% COVID-19-related knowledge was more anxious about performing these procedures when compared to the group with ≤70% COVID-19-related knowledge, *p* < 0.05 (considered significant using the Pearson chi square test). This could be due to the high risk associated with these aerosol producing procedures as the latter stay in the air for long and increase the transmissibility of the virus [21]. Nigerian orthodontic residents and orthodontists regarded recording patients' impressions a highly risky procedure (97.9%). On the other hand, this study showed that most of the respondents were just fairly or not anxious while recording impressions of their patients. Moreover, the anxiety experienced by the respondents in our study while debonding orthodontic brackets corroborated with the results of the previously mentioned study where the respondents (95.8%) considered it a high risk. In the same survey, 62.5% of the respondents believed examination of the patients to be highly risky, which was also reflected in this study where the adequately updated group showed more anxiety while consulting new patients (which includes taking their history and examining them clinically) as compared to the other group with <70% COVID-19-related knowledge [22].

A significant association was found with respondents having >70% COVID-19-related knowledge being more anxious while performing orthodontic procedures themselves when compared to their counterparts. This association was not significant with regard to observing the procedures in the OPD. The same group felt more anxious about contracting the infection at their workplaces, risking their own and their families' health, disturbed patients' appointment's schedule, and completing their cases in time in order to appear for their postgraduate exam. This finding was also reflected in the previous researches where anxiety about risking exposure of the family members to COVID-19 from the work places was the top most concern of the healthcare workers (COVID-19 outbreak, 2020) [23]. On the other hand, a study on health professionals and general population found more anxiety in those participants who lacked COVID-19 knowledge [24]. These findings become all the more important with regard to the quality of orthodontic training program as the completion of at least five orthodontic cases up to a certain degree of excellence is mandatory to sit the postgraduate exams at the end of the four year training program. Orthodontic patients are followed at least once a month for smooth and timely progression of their treatment. Failure to do so could prolong the treatment, affect its quality adversely, lead to more breakages of the orthodontic attachments, and make the patients more prone to caries and periodontal disease. Such a situation could challenge the orthodontic residents' patience and resilience whose prospects of sitting and passing their postgraduate exams are reliant on their knowledge, expertise, and quality of their completed cases. Residents in other specialties too also suffered during the COVID-19 pandemic, which affected their mental health a great deal. More than 90% of the urology residents in France were shown to be stressed due to the pandemic's impact on their work and personal lives [25].

This study also assessed the impact of the pandemic on the residents' anxiety around the disturbed schedule of curricular activities. Most of the residents in both the groups confirmed disturbance in their study schedule. Yet, there was no association found between the level of COVID-19-related knowledge and the anxiety experienced. On the contrary, most of the respondents in both the groups agreed that the social distancing practiced during the pandemic did not jeopardize group studying with fellow residents. Interestingly, a study reported lesser anxiety in students living in dormitories than those living with their parents [26]. Majority of the residents in both the groups believed that the pandemic had made the conducting of routine journal clubs, case presentations, and lectures, a challenge, which could have negative repercussions on the quality of training. Moreover, the suspension of national and international conferences/workshops at that time had limited their exposure to learning. No association was found for these parameters between the two groups, whatsoever. Radiology residents in Canada also reported anxiety due to less on-site time, limited clinical exposure, missed rotations, decreased case volume, and staff-resident interaction during the pandemic [27]. This corroborated with the findings of a survey conducted by the American Confederation of Urology (CAU), stating that 75% of the surgical trainees felt that their training had been completely affected and 80% of them thought that measures should be taken to compensate for the loss incurred [28]. The results of this study showed that the shift to virtual learning was welcomed whole heartedly by the masses as most of the respondents favored it to minimize close human contacts. Previous studies also showed that the best thing to come out of the pandemic was “online/virtual learning”! [29, 30]. Furthermore, in this study, the group with more COVID-19-related knowledge suffered more anxiety around the preparation for theoretical and clinical components of the postgraduate exam.

Finally, the residents were asked about their thoughts revolving around the current status of their training. Majority of the residents in both the groups opted for extension of their training program. Interestingly, more of the residents from the group with ≤70% COVID-19-related knowledge selected this option, 51.8% compared with 39.8% from the group with >70% COVID-19-related knowledge. In a study, 71% of gastroenterology trainees in Canada were concerned about prolonging their training [31]. Furthermore, a minority of the Saudi neurosurgery residents also opined extension of their training to make up for the loss the pandemic had done, while, others felt their planned fellowships would compensate for the latter [32]. To cater to the deteriorating mental health of the residents, the United States of America initiated wellness programs and residents' mental health was monitored through virtual sessions [33]. This was clearly lacking in this part of the world where it could have been a blessing for the frustrated trainees.

Limitations to our study include the 52% responses received across the country, despite constant reminders. This could be due to innumerable survey questionnaires being circulated online at that time and filling each one might have become overbearing for the trainees. Second, the assessment of COVID-19-related knowledge online carried a risk of bias as the respondents could verify their answers through the web while filling the questionnaire. Finally, the study could not get responses from the residents not using smart phones or not accessible through WhatsApp.

## 5. Conclusion

The study started at the time when the third wave of the pandemic peaked in the country, but still gives an insight into the situation faced by the orthodontic postgraduate residents. Today, when the entire world has relaxed its COVID-19-related restrictions, the risk of contracting the disease has still not vanished. This study showed that most of the orthodontic residents were well aware of the COVID-19 infection and the pandemic had adverse effects on their training and mental health that most of them wished for an extension of their training period. Orthodontic residents experienced anxiety at their workplaces across the country and it was shown that those who were more aware about the virus experienced greater anxiety than their counterparts. This study opens the discussion for measures that could be taken to combat the anxiety of the residents, make their workplaces safer in terms of cross infection control, and improve their preparedness, if faced with a similar situation in future.

## Figures and Tables

**Table 1:** Comparison of COVID-19-related knowledge with baseline characteristics (*n* = 171).

Characteristics		*COVID-19 knowledge scores*				*p* value
		*≤70% (n* *=* *83)*		*>70% (n* *=* *88)*		
		*n*	%	*n*	%	
*Age group*	20–29 years	46	55.4	46	52.3	0.91
	30–40 years	37	44.6	42	47.7	

*Gender*	Female	60	72.3	60	68.2	0.55
	Male	23	27.7	28	31.8	

*In which province of the country is your training institute located?*	Baluchistan	7	8.4	2	2.3	0.22
	KPK (Khyber Pakhtunkhwa)	16	19.3	24	27.3	
	Punjab	39	47.0	42	47.7	
	Sindh	21	25.3	20	22.7	

*Which of the following sectors does your training institute belong to?*	Private	28	33.7	40	45.5	0.11
	Public	55	66.3	48	54.5	

*Which year's resident/trainee are you?*	1^st^ year	9	10.8	4	4.5	0.20
	2^nd^ year	14	16.9	13	14.8	
	3^rd^ year	15	18.1	11	12.5	
	4^th^ year	45	54.2	60	68.2	

*Do you think you are updated on the current knowledge and information related to COVID-19?*	Maybe	31	37.3	23	26.1	0.08
	No	10	12.0	6	6.8	
	Yes	42	50.6	59	67.0	

^*∗*^ *p* < 0.05 was considered statistically significant using Pearson chi square test.

**Table 2:** Outcomes on the knowledge regarding COVID-19.

Questions		*n*	%
*Which of the following do you think is the cause of COVID-19?*	SARS-CoV-1	41	24.0
	SARS-CoV-2	109	63.7
	SARS-CoV-3	9	5.3
	SARS-CoV-4	12	7.0

*What do you think is the average incubation period of COVID-19?*	1–2 days	2	1.2
	2–3 days	27	15.8
	5–6 days	95	55.6
	8–9 days	47	27.5

*What is the minimum time period for isolation from the last contact with a confirmed COVID-19 case?*	5–6 days	8	4.7
	7–12 days	35	20.5
	14–15 days	124	72.5
	25–30 days	4	2.3

*What do you know about the minimum social distance required to prevent the spread of COVID-19?*	2 feet	4	2.3
	3 feet	13	7.6
	5 feet	8	4.7
	6 feet	146	85.4

**Table 3:** Outcomes on tests available, treatment options present, and PPE recommendation for COVID-19.

Questions		*n*	%
*What are the primary tests available, to date, for the diagnosis of COVID-19?*	Antibody test	117	68.4
	RT-PCR	155	90.6
	Blood culture	16	9.4
	Sputum culture	28	16.4
	Breath test	11	6.4

*What are the current treatment options present to treat COVID-19 infection?*	Hydroxychloroquine	71	41.5
	Vitamin C	97	56.7
	Remdesivir	92	53.8
	Vitamin D	59	34.5
	Supplemental oxygen	132	77.2
	Monoclonal antibodies	52	30.4
	Mechanical ventilatory support	113	66.1
	Dexamethasone	110	64.3
	Anticoagulants	54	31.6

*Which of these is/are included in the PPE recommendation by WHO for healthcare workers?*	Medical masks	155	90.6
	Respirators	86	50.3
	Face shields	161	94.2
	Eye goggles	148	86.5
	Gown	155	90.6
	Apron	91	53.2
	Gloves	159	93
	Medical mask if tolerated	60	35.1

**Table 4:** Association of anxiety experience by an orthodontist while performing routine procedures and COVID-19 knowledge.

Questions	*COVID-19 knowledge ≤70%*					*COVID-19 knowledge >70%*					*p*value
	N.A	S.A	F.A	V.A	E.A	N.A	S.A	F.A	V.A	E.A	
Changing of ligatures	42 (50.6)	23 (27.7)	13 (15.7)	4 (4.8)	1 (1.2)	49 (55.7)	12 (13.6)	21 (23.9)	2 (2.3)	4 (4.5)	0.08
Changing of arch wires	40 (48.2)	25 (30.1)	14 (16.9)	4 (4.8)	0 (0)	43 (48.9)	18 (20.5)	20 (22.7)	4 (4.5)	3 (3.4)	0.21
Etching with washing and drying with triple syringe and suction	2 (2.4)	16 (19.3)	33 (39.8)	25 (30.1)	7 (8.4)	5 (5.7)	14 (15.9)	19 (21.6)	27 (30.7)	23 (26.1)	<0.01^*∗*^
Bands cementation	22 (26.5)	26 (31.3)	26 (31.3)	6 (7.2)	3 (3.6)	20 (22.7)	18 (20.5)	27 (30.7)	16 (18.2)	7	0.10
Bonding of orthodontic brackets	16 (19.3)	19 (22.9)	34 (41)	9 (10.8)	5 (6)	18 (20.5)	16 (18.2)	22 (25)	16 (18.2)	16 (18.2)	0.03^*∗*^
Impression taking	10 (12)	14 (16.9)	38 (45.8)	16 (19.3)	5 (6)	24 (27.3)	14 (15.9)	26 (29.5)	16 (18.2)	8 (9.1)	0.07
Taking extraoral and intraoral pictures of the patients for record keeping	22 (26.5)	32 (38.6)	19 (22.9)	7 (8.4)	3 (3.6)	32 (36.4)	22 (25)	20 (22.7)	7 (8)	7 (8)	0.26
Removal of bands	21 (25.3)	23 (27.7)	26 (31.3)	7 (8.4)	6 (7.2)	28 (31.8)	15 (17)	27 (30.7)	12 (13.6)	6 (6.8)<	0.42
Debonding using hand piece to remove the adhesive	4 (4.8)	10 (12)	21 (25.3)	30 (36.1)	18 (21.7)	3 (3.4)	4 (4.5)	17 (19.3)	22 (25)	42 (47.7)	<0.01^*∗*^
Debonding using hand instruments to remove the adhesive	7 (8.4)	21 (25.3)	34 (41)	16 (19.3)	5 (6)	15 (17)	22 (25)	22 (25)	17 (19.3)	12 (13.6)	0.08
Separators' insertion	27 (32.5)	28 (33.7)	19 (22.9)	7 (8.4)	2 (2.4)	31 (35.2)	27 (30.7)	17 (19.3)	6 (6.8)	7 (8)	0.53
Insertion and activation of removable appliances	26 (31.3)	24 (28.9)	23 (27.7)	7 (8.4)	3 (3.6)	31 (35.2)	23 (26.1)	20 (22.7)	9 (10.2)	5 (5.7)	0.86
Consultations of the new patients visiting the orthodontic OPD	19 (22.9)	34 (41)	22 (26.5)	6 (7.2)	2 (2.4)	31 (35.2)	17 (19.3)	25 (28.4)	8 (9.1)	7 (8)	0.02^*∗*^

^*∗*^ *p* < 0.05 was considered statistically significant using Pearson chi square test.

**Table 5:** Association of anxiety levels on observing and performing orthodontic procedures with COVID-19 knowledge.

How worried/apprehensive are you?	*COVID-19 knowledge ≤70%*					*COVID-19 knowledge >70%*					*p*value
	N.A	S.A	F.A	V.A	E.A	N.A	S.A	F.A	V.A	E.A	
While observing the orthodontic procedures at your institute's OPD?	15 (18.1)	32 (38.6)	25 (30.1)	8 (9.6)	3 (3.6)	25 (28.4)	21 (23.9)	26 (29.5)	9 (10.2)	7 (8)	0.17
While performing the orthodontic procedures yourself?	10 (12)	27 (32.5)	32 (38.6)	10 (12)	4 (4.8)	8 (9.1)	31 (35.2)	18 (20.5)	20 (22.7)	11 (12.5)	0.02^*∗*^
To be contracting the COVID-19 infection from your OPD/institute and risking yours and your family's health?	3 (3.6)	21 (25.3)	27 (32.5)	14 (16.9)	18 (21.7)	5 (5.7)	17 (19.3)	10 (11.4)	19 (21.6)	37 (42)	<0.01^*∗*^
About disturbed patient appointments due to lockdowns, etc., leading to disturbed tooth movement and possible negative impact on the quality of final treatment results?	3 (3.6)	24 (28.9)	20 (24.1)	17 (20.5)	19 (22.9)	3 (3.4)	11 (12.5)	15 (17)	18 (20.5)	41 (46.6)	<0.01^*∗*^
How do you rate your anxiety level related to the pressure of completing your orthodontic cases in time in order to appear for the final postgraduate exam, especially during the pandemic when the lockdowns have been imposed and the risk still persists?	1 (1.2)	23 (27.7)	18 (21.7)	14 (16.9)	27 (32.5)	5 (5.7)	6 (6.8)	13 (14.8)	20 (22.7)	44 (50)	<0.01^*∗*^
Are you worried about your patients undergoing orthodontic treatment at your training institute, quitting their treatment in between due to the pandemic?	8 (9.6)	18 (21.7)	18 (21.7)	15 (18.1)	24 (28.9)	8 (9.1)	4 (4.5)	17 (19.3)	16 (18.2)	43 (48.9)	0.46

^*∗*^ *p* < 0.05 was considered statistically significant using Pearson Chi square test.

**Table 6:** Association of COVID-19 knowledge with training related academic/curricular activities studied.

Variables		*COVID-19 knowledge*				*p* value
		*≤70%*		*>70%*		
		*n*	%	*n*	%	
Anxiety causing disturbance in study schedule	Yes	51	61.4	52	59.1	0.75
	No	32	38.6	36	40.9	

The need for social distancing jeopardizes activities like group study with other fellow residents	Yes	32	38.6	40	45.5	0.36
	No	51	61.4	48	54.5	

Conducting routine journal clubs, case presentations, and lectures have become difficult and worrisome	Yes	49	59.0	57	64.8	0.44
	No	34	41.0	31	35.2	

Lack of national and international workshops/seminars/conferences is causing limitation in learning and exposure	Yes	46	55.4	59	67.0	0.11
	No	37	44.6	29	33.0	

Apprehensive about disturbed patient appointments, their lack of attendance leading to prolonged treatment duration	Yes	54	65.1	69	78.4	0.052
	No	29	34.9	19	21.6	

It has affected my preparation for the final postgraduate exam, theoretical/clinical	Yes	38	45.8	54	61.4	0.041^*∗*^
	No	45	54.2	34	38.6	

Do you think, to minimize close human contacts, the academic activities related to the postgraduate training, (e.g., lectures, journal club meeting, case presentations) should be done online during this pandemic?	Maybe	18	21.7	14	15.9	0.45
	No	12	14.5	10	11.4	
	Yes	53	63.9	64	72.7	

^*∗*^ *p* < 0.05 was considered statistically significant using Pearson chi square test.

## Data Availability

The data used to support the findings of this study are available from the corresponding author on reasonable request.

## Abbreviations

SPSS: Statistical Package for Social Sciences
PPE: Personal protective equipment
NA: Not anxious
SA: Slightly anxious
FA: Fairly anxious
VA: Very anxious
EA: Extremely anxious
CDC: Centers for Disease Control and Prevention
OPD: Outpatient department
RT-PCR: Real-time polymerase chain reaction
WHO: World Health Organization
SARS CoV: Severe acute respiratory syndrome coronavirus 2
CAU: American Confederation of Urology.
